# Nitroxyl protects H9C2 cells from H/R-induced damage and inhibits autophagy via PI3K/Akt/mTOR pathway

**DOI:** 10.1371/journal.pone.0314500

**Published:** 2025-01-29

**Authors:** Li Li, Zhixin Wang, Yaxuan Lyu, Yanqing Guo

**Affiliations:** 1 Precision Laboratory of Vascular Medicine, Shanxi Cardiovascular Hospital Affiliated Shanxi Medical University, Taiyuan, PR China; 2 Department of Cardiology, Shanxi Cardiovascular Hospital Affiliated Shanxi Medical University, Taiyuan, PR China; Georgia State University, UNITED STATES OF AMERICA

## Abstract

**Background:**

Myocardial ischemia-reperfusion injury (MIRI) is an important complication in the treatment of heart failure, and its treatment has not made satisfactory progress. Nitroxyl (HNO) showed protective effects on the heart failure, however, the effect and underlying mechanism of HNO on MIRI remain largely unclear.

**Methods:**

MIRI model in this study was established to induce H9C2 cell injury through hypoxia/reoxygenation (H/R) in vitro. The cell viability was assessed by cell counting kit-8 assay. The effect of HNO on the apoptosis was detected by flow cytometry. DCFH-DA fluorescent probe method was applied to detect the level of intracellular reactive oxygen species (ROS). The morphology of mitochondria and autophagosomes were observed by transmission electron microscopy. Apoptosis, autophagy and PI3K/Akt/mTOR pathway-related proteins were detected by western blot.

**Results:**

The viability of H9C2 cells was significantly increased in the HNO group. HNO inhibited apoptosis and regulated expressions of key apoptotic protein, including Bax and Bcl-2. HNO reduced ROS levels and alleviated H/R-induced mitochondrial damage. HNO also inhibited autophagy and regulated expressions of key autophagy-related molecules, including LC3II, p62 and Beclin1. Further experiments demonstrated that the effects of HNO were mediated through upregulation of PI3K/Akt/mTOR pathway. Rapamycin reversed the inhibition of HNO on H/R-induced autophagy in H9C2 cells, which abrogated the protective effect of HNO.

**Conclusion:**

This study provided the first evidence that HNO protected H/R-induced cardiomyocytes through inhibiting autophagy via the activation PI3K/Akt/mTOR pathway.

## 1 Introduction

Heart failure (HF) is the terminal stage of various heart diseases [1]. Approximately 64.3 million patients worldwide have HF [2], and the rate is increasing by 2 million yearly, with a poor prognosis and a heavy economic burden on society and families [3, 4]. Myocardial ischemia-reperfusion injury (MIRI) is the most important cause of myocardial injury and HF [5]. Despite the great effort in MIRI research, effective therapeutic strategies remain limited.

Autophagy is a core molecular pathway for preserving cellular and organismal homeostasis. Under normal physiological conditions, autophagy is maintained at inadequate baseline levels and provides nutrients to cells by degrading damaged organelles, misfolded proteins, and pathogens [6, 7]. The role of autophagy in MIRI is paradoxical. While some studies suggest that autophagy is cardio-protective following myocardial infarction, other studies fail to highlight autophagy’s beneficial effect [8, 9]. PI3K/Akt/mTOR pathway, a classical autophagy inhibitory signaling pathway, is crucial in regulating autophagy [10]. Exploring the role and potential mechanisms of autophagy in MIRI may help establish new treatment strategies.

Nitroxyl (HNO), the one electron reduced and protonated form of nitric oxide (NO), displays unique chemistry and pharmacology characteristics distinct from NO [11, 12]. Furthermore, HNO exerts vasodilatory effects in vivo through soluble guanylate cyclase dependent pathways [13], upregulates SERCA2a expression independently of the cAMP/PKA pathway [14], increases mitochondrial activity [15] and promotes calcium ion absorption [16], thereby enhancing cardiomyocyte contractile function. Our previous study illustrated that HNO enhances cardiac function and improves hemodynamics in HF rats [17]. Previous studies on the HNO mechanism have mainly focused on HF. However, the HNO effect on MIRI injury has rarely been reported. In addition, whether HNO is involved in regulating autophagy in cardiomyocyte injury remains poorly understood.

Hence, the HNO effect on H9C2 cells injury induced by hypoxia /reoxygenation (H/R) was investigated in vitro. The HNO regulative effects on the PI3K/Akt/mTOR signaling pathway were also explored intensively. This study provides new insights into the mechanism of HNO on H/R-induced cardiomyocyte injury.

## 2 Materials and methods

### 2.1 Cell culture and treatment

The H9C2 cells (Procell Life Science &Technology Co., Ltd. Wuhan, China) were grown in DEME, including 10% (v/v) fetal bovine (Royacel, Lanzhou, China) serum and 1% (v/v) penicillin streptomycin solution at 37°C in the incubator with 5% CO_2_ and 95% air, and passaged when cell reached about 80% confluence.

### 2.2 H/R cell model establishment

To establish vitro H/R cell model, H9C2 cells were cultured for 24 h in an anoxic incubator containing 95% N_2_ and 5% CO_2_, and maintained in a normal incubator for 12h. The cells were divided into control, H/R, H/R + HNO, H/R + HNO + Rapamycin (H/R + HNO + Rapa), and H/R+ Rapa group. In all model groups, H9C2 cells were pretreated with a culture medium containing 25μmol/L CXL-1002 (donor of HNO, MCE HY-147384) and 80nmol/L rapamycin (MCE HY-10219) and subjected to H/R treatment after replacing the cell culture medium with blood free medium.

### 2.3 Cell viability measurement

The viability of H9C2 cells was performed by using a Cell Counting Kit 8 (CCK-8) assay (HYCCK8-500T, HYCEZMBIO). H9C2 cells were suspended in culture medium and seeded into 96-well plates with a density of 5000 cells per well for 24 h. After the cells in each group were treated accordingly, cells were incubated with 10μl of CCK-8 solution at 37°C for 2 h. The absorbance was analyzed by using a microplate reader at the 450 nm.

### 2.4 Flow cytometric analysis of apoptosis

Annexin V-APC/7-AAD apoptosis detection kit (Jiangsu Kaiji Biotechnology Co., LTD, KGA1026) was used to test apoptosis in H9C2 cell. Furthermore, H9C2 cells at the logarithmic growth stage and in good growth state were inoculated in 6-well plates with 2.5×10^5^ cells per well and cultured overnight at 37°C. The cells were treated differently based on the experimental groups. H9C2 cells were trypsinized using EDTA-free 0.25%, centrifuged at 1,500 rpm for 5 min (G force, 2250×g), and the supernatant was removed. The cells were then re-suspended with PBS. Furthermore, the cells were mixed with 50μl of Binding Buffer and 5μl of 7-AAD for 15 min in the dark. Following this, 450μl of Binding Buffer and 5μl of Annexin V-APC were added and mixed for 15 min in the dark. Subsequently, the percentage of apoptotic cells was determined using a flow cytometer (Beckmancoulter, cytoFLEX).

### 2.5 Detection of reactive oxygen species (ROS)

The cells in the logarithmic growth phase were made into a single-cell suspension with DMEM medium (2.5×10⁵ cells/ml.) and the cells are evenly inoculated into 6-well plates. When the density reaches about 60%, the cells were treated according to the grouping in Section 2.2. The cells are digested and collected using trypsin. According to the instructions of the ROS detection kit (Shanghai Beyotime Biotechnology Co., Ltd.), add 1 ml of DCFH-DA probe diluted with serum-free medium (1:1000) and incubate at 37°C for 20 minutes. Invert and mix every 3–5 minutes to ensure full contact between the probe and the cells. The cells were washed 3 times with serum-free medium to remove excess DCFH-DA, and finally resuspended with 500 μl PBS. The fluorescence intensity of each group is detected by a flow cytometer (Beckman Coulter, cytoFLEX). The fluorescence intensity reflects the ROS content.

### 2.6 Transmission electron microscope

H9C2 cells were washed with PBS and collected. The cells were fixed with 2.5% glutaraldehyde for 4 h, post-fixed in 1% osmic acid for 2h, and dehydrated in graded ethanol (30%, 50%, 70%, 80%, 95%, and 100%). Subsequently, the samples were embedded in Epon at 37°C for 12h and solidified at 60°C for 24 h. The embedding blocks were sliced into 70-nm-thick ultrathin sections on a microtome. Ultrathin sections were sequentially stained with 2% uranyl acetate and 2.6% lead citrate solution for 8 min. The morphology of the mitochondria and autophagosomes was observed using transmission electron microscopy (HITACHI, HT7800).

### 2.7 Western blot

A Radio Immunoprecipitation Assay (RIPA, Meilunbio, MA0151) buffer containing protease inhibitors and phosphatase inhibitors was used for the 30 minute lysis of H9C2 cells on ice. A bicinchoninic acid assay (BCA) analysis kit (GBCBIO, G3522) was utilized for the detection of protein concentrations. The extracted protein supernatant was mixed with 5× loading buffer and boiled in boiling water for 10 min. Equal amounts of total protein were separated by 10% polyacrylamide gel electrophoresis and transferred to polyvinylidene fluoride (PVDF) membrane. Membranes were blocked with 5% skimmed milk in PBST buffer (PBS containing 0.2% Tween 20) at room temperature for 1 h and immunoblotted with primary antibodies overnight at 4°C. Primary antibodies included Bax (1:10000, Wuhan Sanying Biotechnology Co., Ltd., 60627-1-Ig), Bcl-2 (1:3000, Wuhan Sanying Biotechnology Co., Ltd., 26593-1-AP), β-actin (1:2000, Wuhan Sanying Biotechnology Co., Ltd., 66009-1-Ig), LC3 (1:1000, CST, 83506), p62 (1:2000, Wuhan Sanying Biotechnology Co., Ltd., 13916-1-AP), Beclin1 (1:3000, Wuhan Sanying Biotechnology Co., Ltd., 11306-1-AP), mTOR (1:1000, BIOSS, Bs-1992R), p-mTOR(1:1000,CST, 5536), PI3K(1:1000, Affinity, AF6241), p-PI3K(1:1000, CST, AF3241), AKT(1:3000, Wuhan Sanying Biotechnology Co., Ltd., 10176-2-AP), p-AKT(1:10000, Wuhan Sanying Biotechnology Co., Ltd., 66444-1-Ig). The membranes were washed and incubated with HRP-conjugated secondary antibodies (1:10000, Wuhan Boster Biological Engineering Co., Ltd.,) for 2 h at room temperature. Chemiluminescent blot signals were detected through enhanced chemiluminescence (Boster, Wuhan, China) and captured using a chemiluminescence imaging system (Bio-Rad, USA). Image Lab software was used for densitometric analysis. The protein level of β-actin was used as the loading control.

### 2.8 Statistical analysis

Data were assessed using GraphPad Prism software (version 8.3.0). Each cellular experiment was independently conducted at least three times. All experiment data were expressed as mean±standard deviation. Difference between groups were estimated using one-way analysis of variance (ANOVA), followed by Tukey’s post-hoc test. The *p*-value of less than 0.05 was considered statistically significant.

## 3 Results

### 3.1 HNO alleviated H/R-induced H9C2 cells injury

**Fig 1:**
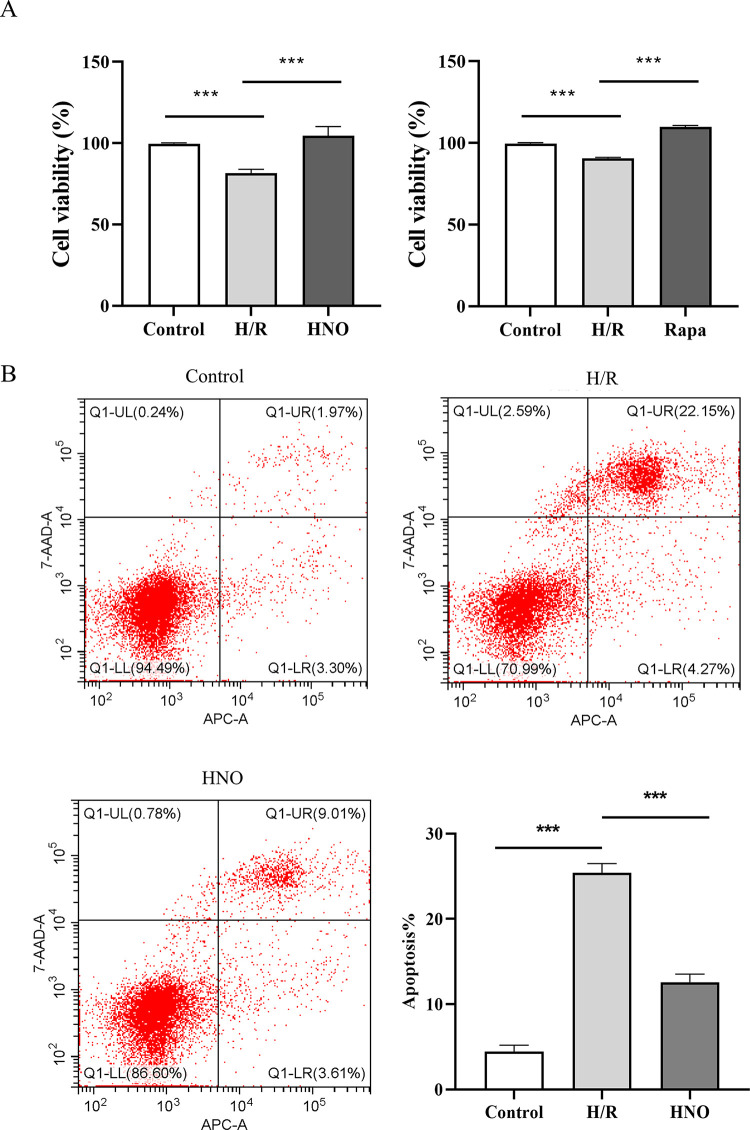
HNO alleviated H/R-induced injury in H9C2 cells. A. Cell viability of H9C2 cells treated with HNO and rapamycin during H/R injury was detected via CCK-8 assay. ***P<0.001. Rapa, rapamycin. B. Flow cytometry was used to evaluate the effect of HNO on H/R-induced apoptosis of H9C2 cells. *** *P*<0.001.

To explore the effect of HNO on H/R-induced H9C2 cell injury, CCK-8 was first used to detect cell activity in each group. Results showed that HNO and rapamycin treatment reversed the attenuation of the viability of H9C2 cells induced by H/R respectively (Fig 1A). Meanwhile, the protective effect of HNO on H/R-induced H9C2 cell injury was investigated using flow cytometry. The cellular apoptotic rate in the H/R group was higher than that in the control group (*P*<0.001). HNO treatment reduced the apoptotic rate (*P*<0.001) (Fig 1B).

### 3.2 HNO inhibited the apoptosis of H/R-induced H9C2 cells

**Fig 2:**
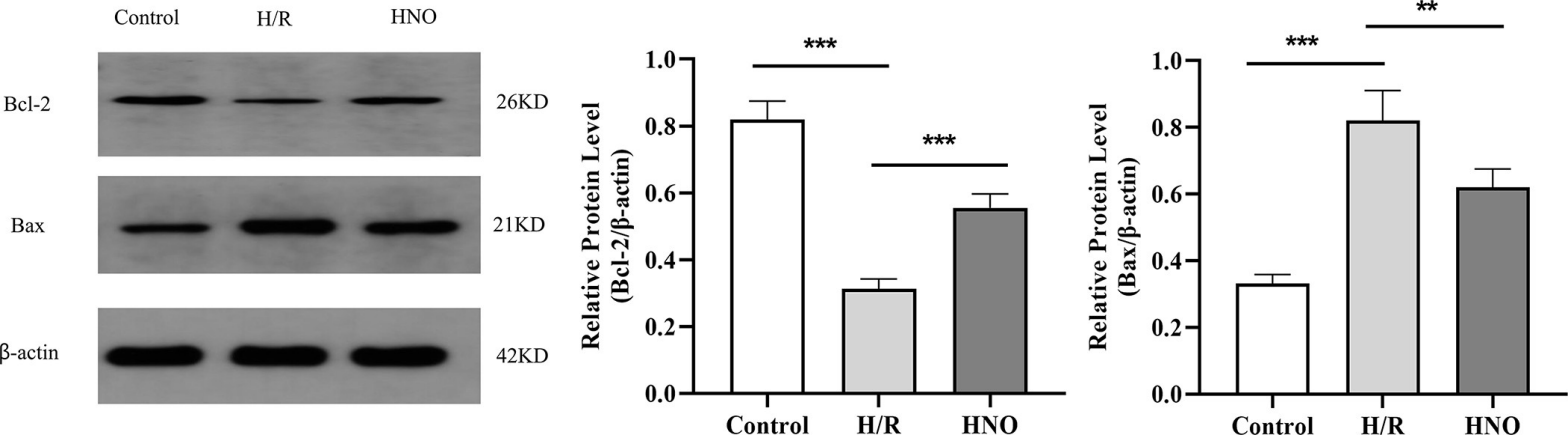
HNO inhibited the apoptosis of H/R-induced H9C2 cells. * *P*<0.05, *** *P*<0.001.

The effect of HNO on apoptosis was further confirmed using Western blot (Fig 2). The expressions of Bax (pro-apoptotic protein) was upregulated in the H/R group compared with the control group (*P*<0.001), whereas downregulated in the HNO group compared with the H/R group (*P*<0.05). Conversely, the expressions of Bcl-2 (anti-apoptotic protein) was downregulated in H/R group compared with the control group (*P*<0.001), whereas upregulated in the HNO group as compared with the H/R group (*P*<0.001).

### 3.3 HNO reduced ROS levels and alleviated mitochondrial damage in H/R-induced H9C2 cells

**Fig 3:**
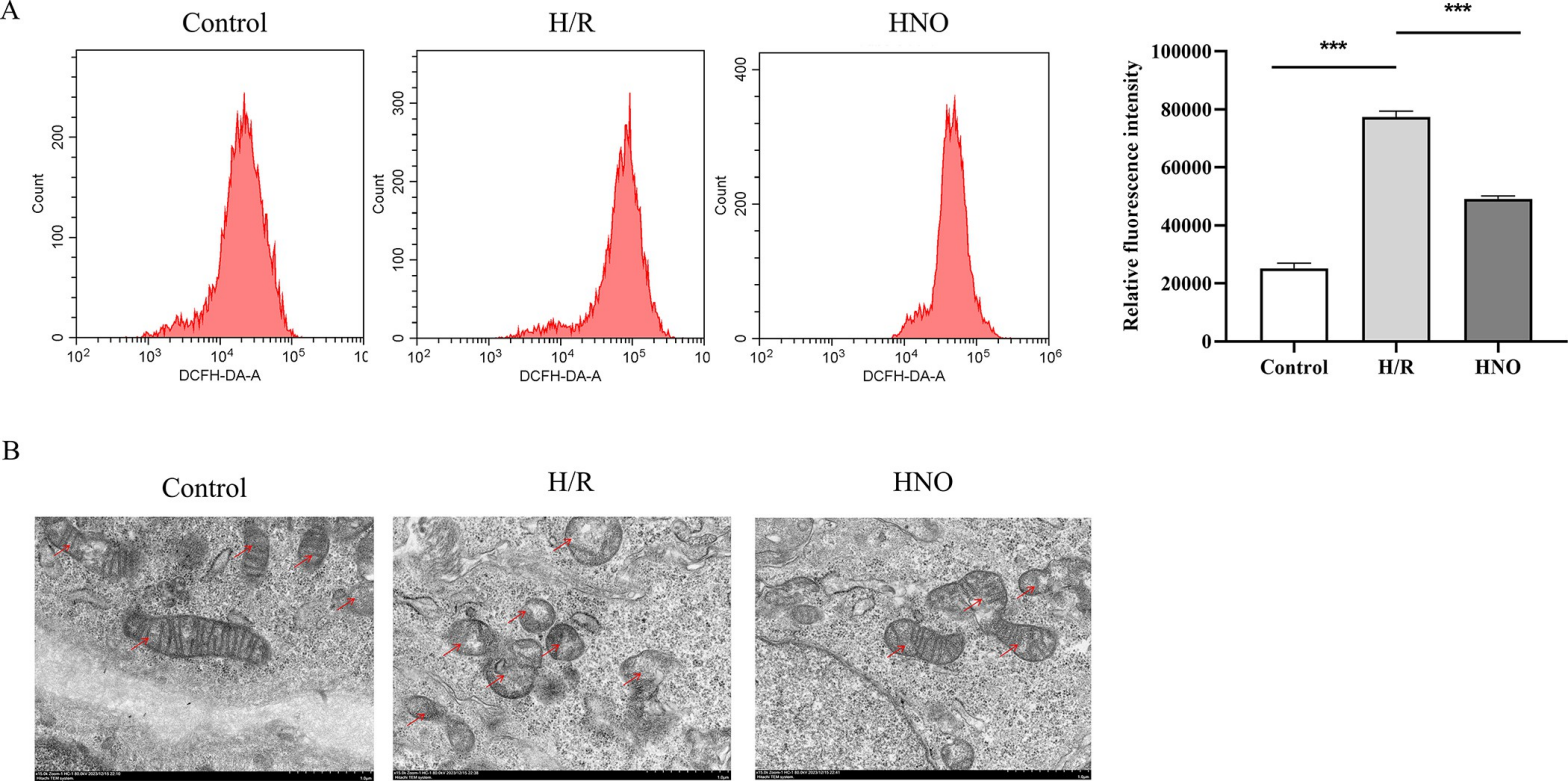
Effect of HNO on ROS and mitochondria. A. Effect of HNO on ROS levels in H/R-induced H9C2 cells. B. Effect of HNO on mitochondrial morphology in H/R-induced H9C2 cells. Red arrow represents the mitochondrial cristae. ***P<0.001.

To evaluate the effect of HNO treatment on oxidative stress in H9C2 cells, ROS levels were detected using the DCFH-DA probe. We found that the H/R-induced ROS upregulation in H9C2 cell was reversed by the treatment of HNO (*P*<0.001) (Fig 3A). Furthermore, the effect of HNO on mitochondrial morphology of H9C2 cells was observed using transmission electron microscopy. The results showed that the H/R group had severe mitochondrial structural damage compared with the control group, with mitochondria becoming round, cristae breaking, and dissolution disappearing. However, mitochondrial structural damage in the HNO group was alleviated, with some mitochondrial cristae breaking (Fig 3B).

### 3.4 Effect of HNO on autophagy in H/R-induced H9C2 cells

**Fig 4:**
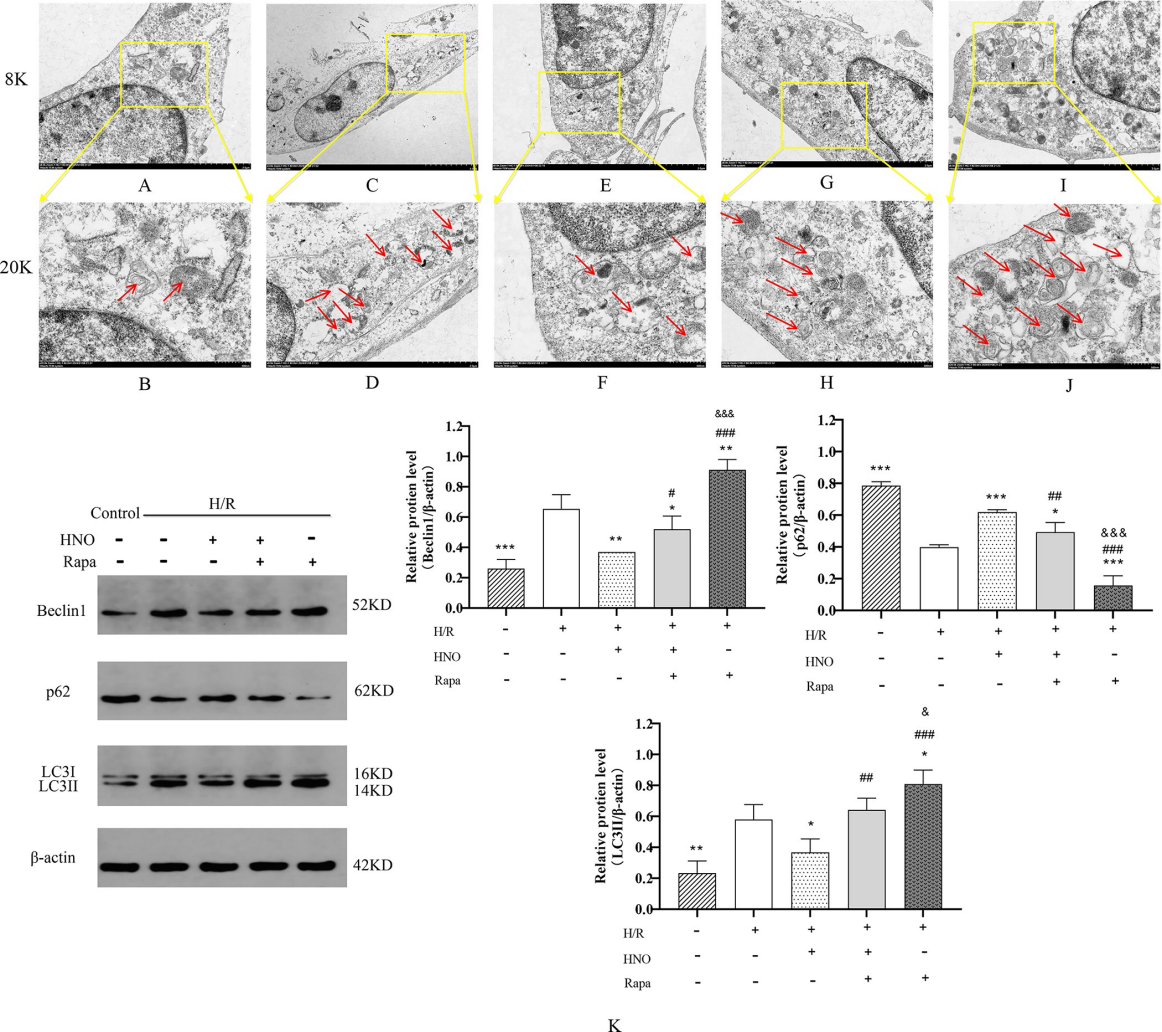
Effect of HNO on autophagy in H/R-induced H9C2 Cells. A-J. Representative transmission electron microscopy images of autophagosomes from different groups. A and B: Control group. C and D: H/R group. E and F: H/R+HNO group. G and H: H/R+HNO+Rapa group. I and J: H/R+Rapa group. Arrows indicate autophagosome. 8K = 8,000×magnification (scale bars represent 2μm), 20K = 20,000×magnification (scale bars represent 500nm). K: HNO regulated the expression of autophagy-related proteins, including LC3II, Belin1 and p62. All data were presented as means ± SD. **P<0.01, ***P<0.001 vs. H/R group. ^#^*P*<0.05, ^##^*P*<0.01, ^###^*P*<0.001 vs. HNO group. ^&^*P*<0.05, ^&&&^*P*<0.001 vs. H/R+HNO+Rapa group.

Transmission electron microscopy was used to observe the effect of HNO on autophagy of H9C2 cells. As shown in Fig 4A–4J, the number of autophagosomes in the H/R group increased significantly compared with the control group, while decreased significantly in the HNO treatment group. Moreover, we examined the effect of HNO on autophagy-associated proteins using western blot. The results showed that levels of p62 were reduced, while levels of Beclin1 and LC3II were increased in the H/R group compared with the control group (*P*<0.001, *P*<0.001, *P*<0.01, respectively). The HNO treatment upregulated p62 expressions and inhibited Beclin1 expressions, suggesting that HNO could partially inhibit autophagy in H/R-induced H9C2 cells (Fig 4K). Notably, Rapamycin is a potent inducer of autophagy. To confirm the mediating mechanism of HNO in H/R-induced H9C2 cell damage, H9C2 cells were pretreated with rapamycin. The results indicated that the number of autophagosomes in the combined treatment group of rapamycin and HNO increased, similar to that in the H/R group (Fig 4A–4J). Besides, rapamycin reversed the inhibition of HNO on autophagy-associated proteins (Fig 4K).

### 3.5 HNO inhibited autophagy by regulating the PI3K/AKT/mTOR pathway

**Fig 5:**
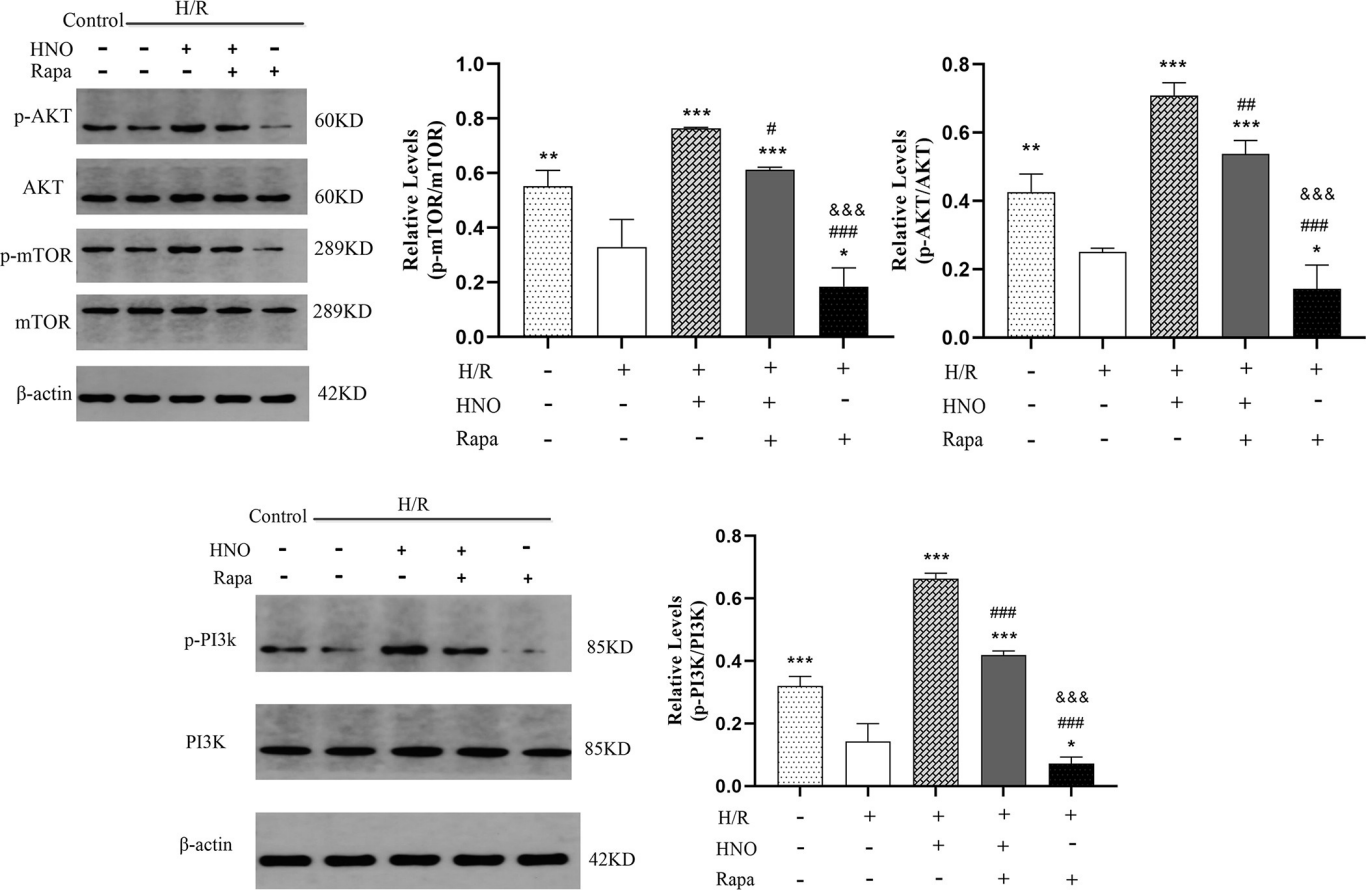
HNO inhibited autophagy by regulating PI3K/AKT/mTOR pathway. **P<0.01, ***P<0.001 vs. H/R group. ^##^*P* <0.01, ^###^*P* <0.001 vs. HNO group. ^&&&^ *P* <0.001 vs. H/R+HNO+Rapa group.

Rapamycin is an autophagy activator and an inhibitor of mTOR kinase. To further investigate whether HNO inhibited autophagy by regulating the PI3K/AKT/mTOR pathway, we pretreated H9C2 cells with rapamycin and detected the protein expression of the PI3K/AKT/mTOR pathway. The results of western blot revealed that expression of p-PI3K, p-AKT, and p-mTOR were reduced in the H/R group compared with the control group (P<0.001, P<0.01, and P<0.01, respectively). HNO upregulated the expression of p-PI3K, p-AKT, and p-mTOR compared with the H/R group (P<0.001). Moreover, co-treatment with HNO and rapamycin reversed their expression to the control group (Fig 5). Notably, the expressions of p-PI3K, p-AKT, and p-mTOR were significantly reduced in the H/R+Rapa group compared with the H/R+HNO (P<0.001) and H/R+HNO+Rapa groups (P<0.001), conversely demonstrating that HNO alleviates H9C2 cells injury by inhibiting autophagy through regulating PI3K/Akt/mTOR pathway.

## 4 Discussion

HNO is the one-electron reduced form of NO [18] that enhances myocardial contractility, improves hemodynamic effects, and inhibits myocardial hypertrophy. It is widely used in HF treatment [19–22]. However, the effect and underlying mechanisms of HNO on MIRI remain poorly understood. Apoptosis is a critical cellar event of MIRI injury related to regulating apoptosis-relayed genes [23]. Our study established an MIRI model in H9C2 cells induced by H/R and found that HNO increased cell activity and inhibited cell apoptosis, suggesting that HNO treatment could alleviate H/R-induced H9C2 cell damage. Overexpression of Bax, a member of the BCL-2 family, accelerates the apoptosis rate [24]. The Bcl-2, an inhibitor factor of apoptosis, is expressed in the infarcted myocardium organ but not in non-infarcted tissue [25]. Mitochondria are the sites of cell energy metabolism and an important way to regulate cell apoptosis [26]. Numerous ROS are known to be produced during MIRI [27]. In addition, overexpression of Bcl-2 increased the permeability of calcium ions and inhibited the production of mitochondrial ROS [28]. This study demonstrated that Bax expression was downregulated, and Bcl-2 expression was upregulated in the HNO group compared with the H/R group. HNO regulated calcium cycling-related proteins and promoted calcium ion reabsorption by cardiomyocytes, thereby reducing oxidative stress levels [29]. Our study found that ROS levels in the HNO group were significantly decreased, and the mitochondrial structural damage was alleviated, suggesting that HNO may play an anti-apoptotic role through the mitochondria- dependent pathway. Moreover, paralleling Daniele et al.’s [30] studies, the protective effect of HNO on H/R-induced H9C2 cell damage may be related to the reduction of ROS levels.

Autophagy is the enzymatic degradation of proteins, fats, nucleic acids, and whole organelles in lysosomes. Typically, autophagy serves as a cell survival mechanism and maintains the structure and biological functions of cardiomyocytes [31]. However, it was found to increase significantly in the MIRI environment. Furthermore, LC3, Beclin 1, and p62 are autophagy marker molecules [32]. Notably, Beclin1 and LC3 initiate autophagy by interacting with ATG3/ATG7 and other autophagy effectors [33]. Besides, p62 is a well-characterized autophagy receptor that drives autophagosome formation through spatial membrane gathering mode [34]. The regulatory role of autophagy in cardiomyocyte injury remains controversial. Autophagy acts as a housekeeper to clear damaged organelles, such as broken mitochondria, remove misfolded proteins, eliminate intracellular pathogens, and recycle cellular components [35]. However, studies have highlighted that autophagy promotes cell death and myocardial remodeling by excessive self-digestion of normal cellular components [36, 37]. In this study, p62 expression was increased, while Beclin1 and LC3II levels were reduced in the HNO group compared with the H/R group, indicating that autophagy was inhibited following HNO treatment. We further verified whether HNO was involved in H/R-induced autophagy in H9c2 cells using rapamycin. Co-treatment of rapamycin and HNO significantly reversed the inhibition of HNO on autophagy-associated proteins, suggesting that HNO alleviated H/R-induced myocardial injury by inhibiting autophagy.

The PI3K/Akt/mTOR is a widely studied upstream signaling pathway for autophagy. Evidence has revealed that the PI3K/Akt/mTOR pathway is crucial in MIRI [38–40]. The effect of HNO on the upstream signaling pathway of autophagy was further investigated to reveal its potential protective mechanism against H/R damage. Ischemia and hypoxia activate the initiation factor PI3K of PI3K/AKT/mTOR and promote AKT activation [41, 42]. The Akt is a central signal transmitter responsible for cellular metabolism and stress response in the PI3K/Akt/mTOR pathway. Activated AKT can activate mTOR, a target of rapamycin. Furthermore, mTOR is a negative regulator of autophagy, with phosphorylated mTOR inhibiting autophagy [43]. In this study, expression levels of PI3K, Akt, and mTOR proteins were upregulated in the HNO group compared with the H/R group, and the PI3K/AKT/mTOR pathway was activated. Notably, rapamycin combined with HNO treatment canceled HNO activation on the PI3K/Akt/mTOR pathway, conversely verifing that HNO protects cardiomyocytes from H/R damage by inhibiting autophagy through an activation PI3K/AKT/mTOR pathway.

The BH3 domain of Beclin1 has been found to interact with Bcl-2 and the disruption of the interaction between these two molecules may lead to the disruption of the balance between autophagy and apoptosis [44]. However, neither previous studies nor our current study has determined whether HNO mediates the crosstalk between apoptosis and autophagy. We will use ABT199, a specific Bcl-2 inhibitor, to further verify whether HNO mediates crosstalk of apoptosis and autophagy during MIRI in future study. Moreover, research has shown that the Bcl-2 has a regulatory effect on mitochondrial membrane permeability. Therefore, the impact of HNO on mitochondrial function will also be a key focus of future research. However, this study has some limitations. First, the results from cell studies were not verified or extended on animal models because in vitro models cannot fully simulate the complex physiological environment in vivo. Second, the hypoxia/reoxygenation model cannot fully simulate intracellular acidity and glucose deficiency in myocardial reperfusion injury. Finally, autophagy inhibitors were not used to compare the ability of HNO to inhibit autophagy. Therefore, a rat model of oxygen-glucose deprivation and autophagy inhibitors will be used to deeply explore the mechanism of HNO on MIRI in future studies.

## 5 Conclusion

In conclusion, this study provides the first evidence that HNO attenuated H/R-induced injury in H9C2 cells and the underlying mechanisms may involve alleviating apoptosis through the mitochondria-dependent pathway and inhibiting autophagy via the activation the PI3K/Akt/mTOR pathway. The protective effect of HNO on H/R-induced H9C2 cells may provide new insights into MIRI treatment.

## Supporting information

S1 Raw images (PDF)
