# Supplementary figures and images for: Nitroxyl protects H9C2 cells from H/R-induced damage and inhibits autophagy via PI3K/Akt/mTOR pathway
Source: PLoS One. 2025 Jan 29;20(1):e0314500. doi: 10.1371/journal.pone.0314500 (PMC11778757; doi:10.1371/journal.pone.0314500)

**Figure 2**

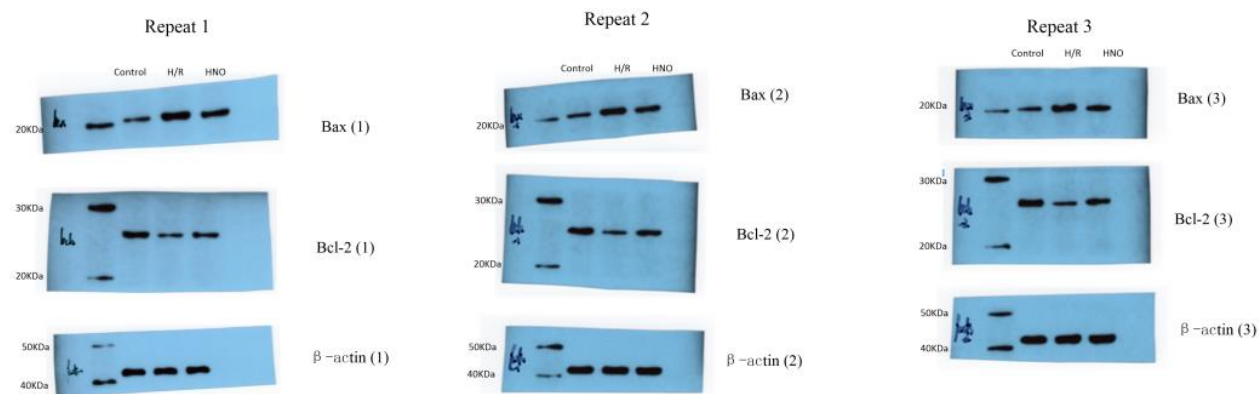

**Figure 4**

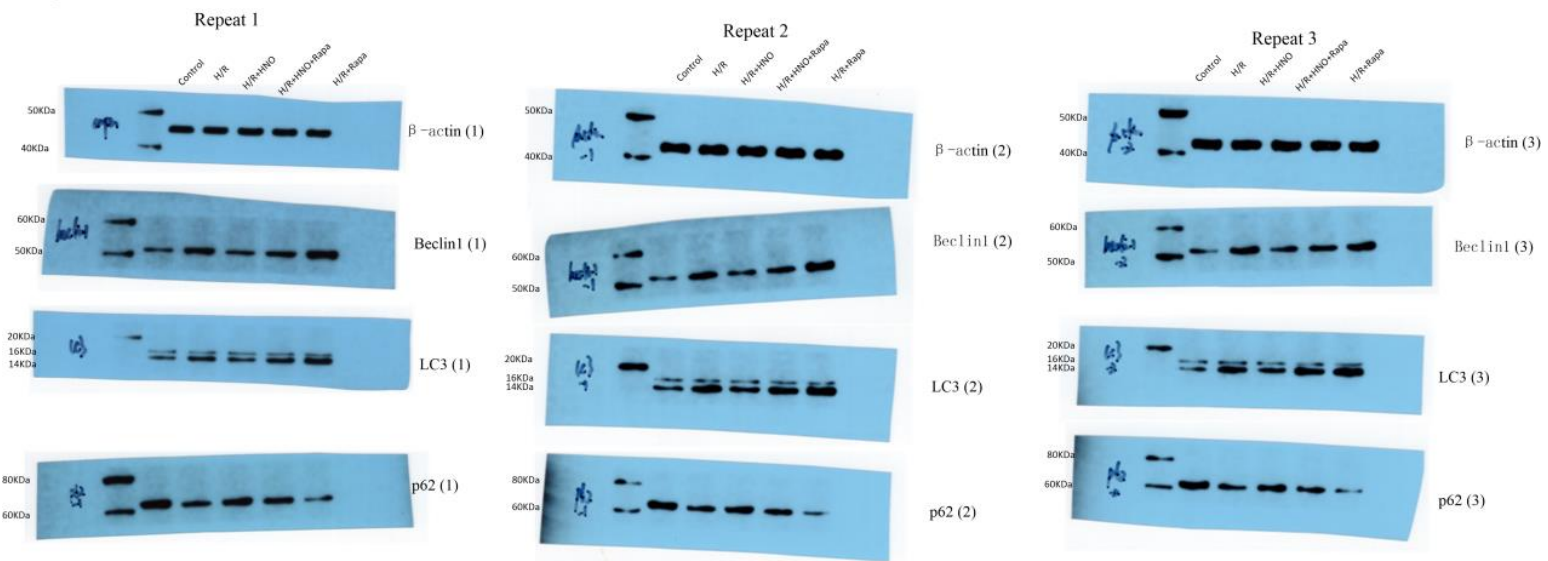

**Figure 5**

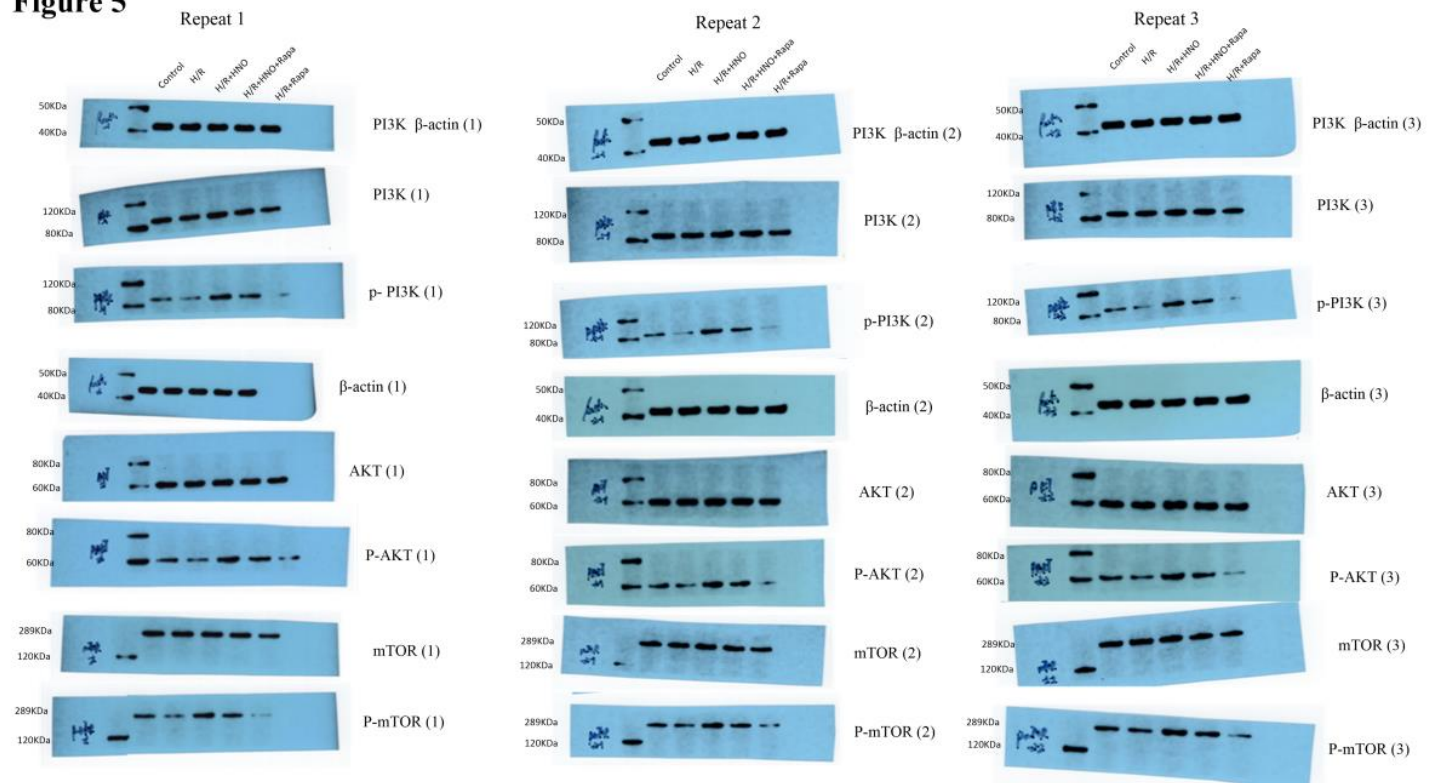

Supplement: S1 Raw images — (PDF) [file pone.0314500.s001.pdf]
